# Selective Isolation and Screening of Actinobacteria Strains Producing Lignocellulolytic Enzymes Using Olive Pomace as Substrate

**DOI:** 10.15171/ijb.1278

**Published:** 2017-03

**Authors:** Lamia Medouni-Haroune, Farid Zaidi, Sonia Medouni-Adrar, Sevastianos Roussos, Samia Azzouz, Véronique Desseaux, Mouloud Kecha

**Affiliations:** ^1^ Laboratoire de Microbiologie Appliquée, Faculté des Sciences de la Nature et de la Vie, Université de Bejaia, 06000 Bejaia, Algérie.; ^2^ Département des Sciences Alimentaires, Faculté des Sciences de la Nature et de la Vie, Université de Bejaia, 06000 Bejaia, Algérie.; ^3^ Laboratoire de Biomathématiques, Biophysique, Biochimie, et Scientométrie (L3BS), Faculté des Sciences de la Nature et de la Vie, Université de Bejaia, 06000 Bejaia, Algérie.; ^4^ Equipe Eco technologies et Bioremédiation, Faculté St Jérome, Campus Etoile, Aix Marseille Université & Université Avignon; IMBE UMR CNRS-7263/IRD-237, Case 421, 13397 Marseille cedex 20, France.; ^5^ Institut des Sciences Moléculaires de Marseille, Faculté des Sciences et Techniques, St Jérome, Biosciences UMR CNRS 6263. Université Paul Cézanne, 13397 Marseille Cedex 20, France.

**Keywords:** Actinobacteria, Lignocellulolytic enzymes, Olive pomace, Submerged fermentation

## Abstract

**Background:**

Olive pomace, as the main by-product of the olive oil industry, is recently recycled as fermentation substrate for enzyme production.

**Objectives:**

Actinobacteria isolates were separated from an Algerian soil under olive pomace cultivation and were evaluated for their lignocellulolytic enzymes production.

**Materials and Methods:**

Isolates of Actinobacteria were separated from soils around oil mills using four isolation media, among them three were enriched by olive pomace. The isolates were screened for their cellulolytic, xylanolytic and ligninolytic activities. Isolates with potential of producing lignocellulose-degrading enzymes were selected under submerged fermentation based olive pomace.

**Results:**

Ninety isolates of Actinobacteria were separated from soil samples. M3 medium (raw pomace autoclaved alone) was the best isolation medium (68 strains), whereas, the soil from oil mill with continuous system (S1) led to separation of 52 strains. Among the 90 isolates, 82 were shown promising enzyme activity, 19 isolates were presented the largest zone diameter (<30 mm). S1M3I and S1M3II isolates were exhibited the highest values.

**Conclusions:**

Olive pomace with medium low cost and high titers of enzymes can be valorized by culture of Actinobacteria to produce lignocellulolytic enzymes for industrial applications.

## 1. Background

Actinobacteria, highly abundant filamentous Gram positive bacteria are ubiquitously present in all natural substrates and soil (1-2). Actinobacteria are involved in recycling hard-to-degrade organic matter such as cellulose, cell wall matrix polysaccharides and lignin (3-4).

Use of low-cost residues from agro-industries, as substrates for growing microorganisms, may constitute an interesting alternative in enzyme industry (4). According to the National Agency for Development of Investment (ANDI, Algeria), 87500 tons of olive pomace are being produced and discarded that can act as pollutant in near future (5). Olive pomace consists of lingnocellulosic matrix with phenolic compounds, uronic acids, and oily residues and may represent an important alternative source for enzymatic processes and biofuel production (4). However, studies dealing with lignocellulolytic production by Actinobacteria using olive pomace residues are rare.

Here, Actinobacteria isolates from an Algerian soil under olive pomace cultivation were separated, and the production of carboxymethyl cellulases (CMCases), xylanases and laccases were evaluated qualitatively and quantitatively.

## 2. Materials and Methods

### 2.1. Collection of lignocellulosic Samples

Olive pomace was collected from oil mill (Algeria), dried at 22°C for 3 weeks, ground to a fine powder, passed through sieves (ᴓ ≤ 75 µm and ᴓ ≤ 1 mm). One portion of the sieved pomace was pretreated by hexane to remove lipids.

### 2.2. Soil Samples and Microorganisms

The samples were taken from the soil of natural discharge of three oil mills; S1: continuous system (annual activity), S2: traditional system (abandoned for 20 years), and S3: traditional system (annual activity). Actinobacteria from soils were isolated using standard dilution plate method (6) on four different media: M2 (Starch Casein Agar medium) containing 10 g.L^-1^ soluble starch, 0.3 g.L^-1^ casein, 1 g.L^-1^ glucose, 2 g.L^-1^KNO3, 2 g.L^-1^ K_2_HPO_4_, 2 g.L^-1^ NaCl, 0.05 g.L^-1^ MgSO_4_.7H2O, 0.02 g/L^-1^ CaCl_2_, 0.01 g.L^-1^ FeSO_4_.7H_2_O, 18 g.L^-1^ agar, pH 7.2 and autoclaved at 120°C. 20 min^-1^) (7).

M1 containing: 8 g.L^-1^ raw pomace powder (ᴓ=75µm), 2 g.L^-1^ soluble starch, 0.3 g.L^-1^ casein, 1 g.L^-1^ glucose, 2 g.L^-1^ KNO_3_, 2 g.L^-1^ K_2_HPO_4_, 2 g.L^-1^ NaCl, 0.05 g.L^-1^ MgSO_4_.7H_2_O, 0.02 g.L^-1^ CaCl_2_, 0.01 g.L^-1^ FeSO_4_.7H_2_O, 18 g.L^-1^ agar, pH 7.4 and then sterilized.

M3 and M4 containing: 8 g.L^-1^ raw pomace powder (ᴓ=75 µm), and 8 g.L^-1^ pretreated pomace powder (ᴓ=75 µm), respectively (Sterilized alone), and aqueous phase containing: 2 g.L^-1^ soluble starch, 0.3 g.L^-1^ casein, 1 g.L^-1^ glucose, 2 g.L^-1^ KNO_3_, 2 g.L^-1^ K_2_HPO_4_, 2 g.L^-1^NaCl, 0.05 g.L^-1^ MgSO^4^.7H_2_O, 0.02 g.L^-1^ CaCl_2_, 0.01 g.L^-1^ FeSO4.7H_2_O, 18 g.L^-1^ agar, pH 7.8 and then sterilized. Rifampicin (2.5 mg.mL^-1^) and amphotericin B (75 mg.mL^-1^) were added to all media in order to inhibit bacterial and fungal contaminations, respectively (6). Plates were incubated at 28°C for 7d. Colonies showing an Actinobacteria-like appearance were puriﬁed and selected based on the Gram staining.

### 2.3. Screening of CMCase, Xylanase and Laccase Producers

A preliminary qualitative analysis was conducted by carboxymethyl cellulose (CMC) and xylan containing agar plates method described by Boroujeni *et al*. (1) and guaiacol containing agar plates method described by Lu *et al*. (8).

### 2.4. Submerged Fermentation

Submerged fermentation was carried out in Erlenmeyer flasks (250 mL) containing 50 mL of the production medium containing: 30 g.L^-1^ raw olive pomace powder (ᴓ ≤ 1 mm), 3 g.L^-1^ (NH_4_)_2_SO_4_, 1.2 g.L^-1^ NaNO_3_, 1.5 g.L^-1^ KH_2_PO_4_, 3 g.L^-1^ K_2_HPO_4_, 0.2 g.L-MgSO_4_7H_2_O, 0.05 g.L^-1^ CaCl_2_, 0.01 g.L^-1^ MnSO_4_.7H_2_O, 0.001 g.L^-1^FeSO_4_.7H_2_O, 0.05 g.L^-1^ yeast extract, pH 7.4. The flasks were autoclaved (olive pomace alone), inoculated with three agar disks (ᴓ= 6 mm) taken from the 7 d old stock culture and incubated at 30°C for 6 d on a rotary shaker at 150 rpm. The crude enzyme preparations were obtained by centrifugation (12,857 ×*g* for 20 min) and used for enzyme assay.

### 2.5. Measurement of CMCase, Xylanase and Laccase Activities

Xylanase and CMCase activities were determined by measuring the release of reducing sugar according to Tuncer *et al*. (9). Laccase activity was assayed according to Criquet *et al*. (10). The results were expressed in units (U), where U is defined as the amount of enzyme required to liberate 1µM of xylose, glucose and quinone per min.

### 2.6. Statistical Treatment

One-way analysis of variance (ANOVA) was used to analyze data and multiple pair-wise comparisons were performed by the Tukey test using Xlstat® software.

## 3. Results

**Figure 1:**
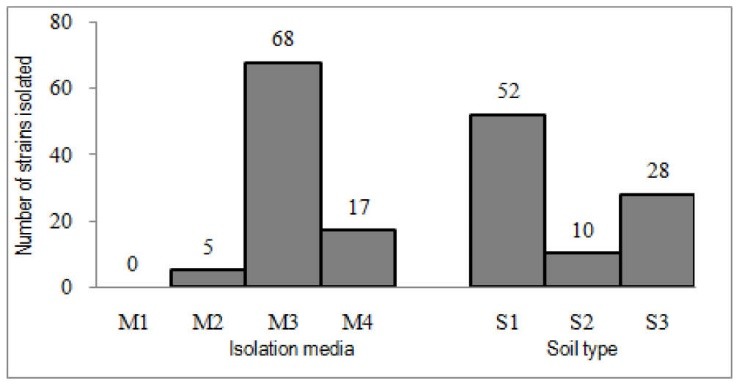
Number of Actinobacteria isolated from diff erent soils (S1, S2 and S3) and ondiff erent media (M1, M2, M3 and M4).

After processing of soil samples, a total of 90 Actinobacteria strains were isolated. Most of the isolates were separated on M3 medium (68 isolates) and negative results belong to M1 (Fig. 1). The S1soil gave 52 isolates (Fig. 1), higher than S2 and S3 (the least number of isolates).

Among the 90 isolates, 82 showed promising enzyme activities (CMCase, xylanase and laccase) with diameter zones varying between 19 mm and 53 mm (Table 1). Nineteen isolates (1), with diameters greater than 30mm, were the most aggressive types. The isolates were further characterized by carrying out enzymatic assays using CMC and xylan as substrates. Furthermore, 3 isolates giving promising enzymatic activities were tested using syringaldazine. All tested isolates exhibited activities, comprised between 1.44-0.31 U.mL^-1^ for CMCase, 6.65-0.79 U.mL^-1^ for xylanase and 5.63 x 10^-3^-2.15 x 10^-3^U.mL^-1^ for laccase (Table 2; p <0.05). The best results were obtained with S1M3I and S1M3II strains for all enzyme activities studied.

**Table 1:** Screening for lignocellulolytic enzymes produced by different Actinobacteria isolates

**Numberof isolates**	**CMCase**	**Xylanase**	**Laccase**
8	-	-	-
11	+	-	-
19	-	+	-
13	+	+	-
20	++	-	-
16	++	+	-
2	+++	++	Pre.
1	+++	+++	Pre.

Pre.: Presence of activity - : No activity, + <30 mm, ++ (30-40 mm) and +++ >40 mm.

**Table 2:** Lignocellulolytic enzyme activities of the selected Actinobacteria strains.

**Isolates**	**CMCase activity** **(U.mL** ^-1^ **)**	**Xylanase activity** **( U.mL** ^-1^ **)**	**Laccase activity** **(10** ^-3^ ** U.mL** ^-1^ **)**
S1M3I	1.44 ± 0.01^a^	6.65 ± 0.16 ^a^	3,42 ± 0.18 ^b^
S1M3II	1.42 ± 0.04 ^a^	5.40 ± 0.42 ^b^	5.63 ± 0.38 ^a^
S1M3III	0.57 ± 0.00 ^g^	2.97 ± 0.10 ^c^	2.15 ± 0.14 ^c^
S3M4I	0.67 ± 0.04^f^	1.82 ± 0.75^d^	-
S3M3I	0.95 ± 0.04 ^c^	2.08 ± 0.03 ^d^	-
S1M3IV	0.89 ± 0.04 ^d, e^	1.79 ± 0.05^d^	-
S1M3V	1.06 ± 0.02 ^b^	0.84 ± 0.06^h^	-
S1M3VI	0.80 ± 0.05 ^e^	1.17 ± 0.12 ^f^	-
S3M3II	0.66 ± 0.04 ^f^	1.16 ± 0.04 ^f^	-
S3M3III	0.56 ± 0.01 ^g, h^	1.21 ± 0.06 ^e, f^	-
S1M3VII	0.53 ± 0.02 ^h^	1.12 ± 0.07^g^	-
S3M3IV	0.50 ± 0.01 ^h^	0.79 ± 0.003 ^h^	-
S1M3VIII	0.68 ± 0.02^f^	0.80 ± 0.02 ^h^	-
S3M2I	0.53 ± 0.08 ^h^	0.93 ± 0.01 ^h^	-
S3M3V	0.58 ± 0.04 ^g, h^	1.40 ± 0.06 ^e^	-
S3M3VI	0.94 ± 0.01 ^c, d^	1.06 ± 0.04 ^g, h^	-
S3M3VII	0.31 ± 0.03^i^	0.97 ± 0.09 ^h^	-
S3M4II	1.03 ± 0.02 ^b, c^	1.16 ± 0.06 ^f, g^	-
S2M4I	0.56 ± 0.03 ^g^	1.61 ± 0.04 ^d, e^	-

No measured Diff erent letters in same column indicate significant diff erence (p <0.05). Results are ranked in ascending order: a>b>c>d>e>f>g>h>i.

## 4. Discussion

Actinobacteria isolates were separated from soil around olive oil mill. It was expected that due to the presence of decomposing olive pomace, the main bacterial species have the capability of producing enzyme that were able to degrade lignocelluloses. The higher number of isolates obtained on M3 medium compared to M2 and M4 can be explained by the fact that the isolation medium has the same characters as the natural environment of sampling. Indeed, Vance and Chapin (11) reported that microbial growth is influenced by the characteristics of its natural environment, that depends upon the chemistry of the organic matter, soil moisture, soil temperature and physical access of enzyme(s) to substrate(s).

Autoclaving of olive pomace, in aqueous medium (the case of M1 medium) induces extraction and diffusion of phenolic compounds in the medium, causing modifications of its physical and chemical characteristics. An increase in temperature increases the efficiency of the extraction since heat render the cell walls permeable (12) and decreases the viscosity of the solvent (13). On the other hand, several studies have demonstrated that polyphenols are responsible for phytotoxic and antimicrobial actions (12,14).

The difference obtained between the two soils from traditional oil mills (S2 and S3) is due to the time given to the natural fauna from soil to degrade the olive pomace (15) and induction of microflora formation(16), among other Actinobacteria. According to Kuzyakov *et al*. (17) very long periods would be necessary to obtain measurable soil transformations. Soils with long-term exposure to contamination with organic compounds have been shown to have structural and functional microbial communities with the ability to adapt and grow under these conditions (2). Indeed, the oil mills with continuous system produced exhausted pomace, unlike to the traditional ones, so less toxic compound (polyphenol) were formed and fatty acids were released by the degradation of lipids in the soil (5).The lipid hydrolysis releases acids, which causes a decrease in soil pH (13). Lipids, organic acids and mostly phenols are considered responsible for the phytotoxicity, representing a severe risk of water and soil pollution (2).

The production of enzymes was tested by fermentation in a submerged medium using olive pomace. At the present time, there is no citation in the scientific literature describing the use of olive pomace for the production of hydrolases (CMCases and xylanases) and oxidases (laccase) enzymes by the Actinobacteria. Grigorevski *et al*. (18) reported CMCase activity of 0.15 U.mL^-1^ by cultivation of *Streptomyces drozdowiczii* on corn steep liquor. Sharma and Bajaj (19) reported xylanase activity of 2.21 U.mL^-1^ by cultivation of *Streptomyces* sp. CD3 on wheat bran. Laccase activity was mainly reported for fungi. Lakhtar *et al*. (20) reported laccase activity of 0.2 U.mL^-1^ by cultivation of *Lentinula edodes* on olive mill waste water.

The results obtained suggest that Actinobacteria are a good producer of cell-wall degrading enzymes using olive pomace as a carbon source which can be valorized by submerged culture. Considering the ratio between the high titers of enzymes produced and the low olive pomace cost and the fact that is a very abundant agricultural residue in Algeria.

## Acknowledgements

The research was supported by Faculty of Nature and Life Sciences, University of Bejaia-Algeria. We thank Professor S. Roussos for the training in the IMBE and ISM2, Marseille (France).

## References

[1] Boroujeni ME, Das A, Prachanthi K, Suryan S, Bhattacharya S (2012). Enzymatic screening and random amplified polymorphic DNA fingerpinting of soil Streptomycetes isolated from wayanad district in Kerala. Ind J Biol Sci.

[2] Polti MA, Aparicio JD, Benimeli CS, Amoroso MJ (2014). Simultaneous bioremediation of Cr(VI) and lindane in soil by actinobacteria. Int Biodeterior Biodegrad.

[3] Aparicio JD, Sola MZS, Benimeli CS, Amoroso MJ, Polti MA (2015). Versatility of Streptomyces sp M7 to bioremediate soils co-contaminated with Cr(VI) and lindane. Ecotoxicol Environ Saf.

[4] Macedo EP, Cerqueira CLO, Souza DAJ, Bispo ASR, Coelho RRR, Nascimento RP (2013). Production of cellulose–degrading enzyme on sisal and other agro-industrial residues using a new brazilian actinobacteria strain Streptomyces sp SLBA–08. Braz J Chem Eng.

[5] Pagnanelli F, Viggi CC, Toro L (2010). Development of new composite biosorbents from olive pomace wastes. Appl Surf Sci.

[6] Thakur D, Yadav A, Gogoi BK, Bora TC (2007). Isolation and screening of Streptomyces in soil of protected forest areas from the states of Assam and Tripura, India, for antimicrobial metabolites. J Med Mycol.

[7] Abdulla H, May E, Bahgat M, Dewedar A (2008). Characterisation of actinomyetes isolated from ancient stone and their potential for deterioration. J Microbiol.

[8] Lu L, Zeng G, Fan C, Ren X, Wang C, Zhao Q, Zhang J, Chen M, Chen A, Jiang M (2013). Characterization of a laccase–like multicopper oxidase from newly isolated Streptomyces sp C1 in agricultural waste compost and enzymatic decolorization of azo dyes. Biochem Eng J.

[9] Tuncer M, Ball AS, Rob A, Wilson MT (1999). Optimization of extracellular lignocellulolytic enzyme production by a thermophilic actinomycete Thermomonospora fusca BD25. Enzyme Microb Technol.

[10] Criquet S, Tagger S, Vogt G, Iacazio G, Le Petit J (1999). Laccase activity of forest litter. Soil Biol Biochem.

[11] Vance ED, Chapin III FS (2001). Substrate limitations to microbial activity in taiga forest floors. Soil Biol Biochem.

[12] Zam W, Bashour G, Abdelwahed W, Khayata W (2012). Effective extraction of polyphenols and proanthocyanidins from pomegranate’s peel Int J Pharm Pharm. Sci.

[13] Yang YC, Yang ZW, Zhang ZH, Li J, Zu YG, Fu YJ (2013). Effect of acid hydrolysis in the microwave–assisted extraction of phenolic compounds from Geranium sibiricum Linne with the guidance of antibacterial activity. J Med Plants Res.

[14] Barbera AC, Maucieri C, Cavallaro V, Ioppolo A, Spagna G (2013). Effects of spreading olive mill wastewater on soil properties and crops , a review. Agric Water Manage.

[15] Birkhofer K, Diekotter T, Boch S, Fischer M, Muller J, Socher S, Wolters V (2011). Soil fauna feeding activity in temperate grassland soils increases with legume and grass species richness. Soil Biol Biochem.

[16] Kuzyakov Y, Blagodatskaya E (2015). Microbial hotspots and hot moments in soil: Concept and review. Soil Biol Biochem.

[17] Kuzyakov Y, Bogomolova I, Glaser B (2014). Biochar stability in soil: Decomposition during eight years and transformation as assessed by compound–specific 14C analysis. Soil Biol Biochem.

[18] Grigorevski de Lima AL, Pires do Nascimento R, da Silva Bon EP, Coelho RRR (2005). Streptomyces drozdowiczii cellulase production using agro-industrial by products and its potential use in the detergent and textile industries. Enzyme Microb Technol.

[19] Sharma P, Bajaj Bk (2005). Production and Partial characterization of alkali–tolerant xylanase for an alkalophilic Streptomyces sp CD3. J Sci Ind Res.

[20] Lakhtar H, Ismaili-Alaoui M, Philippoussis A, Perraud-Gaime I, Roussos S (2010). Screening of strains of Lentinula edodes grown on model olive mill wastewater in solid and liquid state culture for polyphenol biodegradation. Int Biodeterior Biodegrad.

